# Slow Poisoning by Lead

**Published:** 1851-07

**Authors:** 


*Slow Poisoning by Lead.*—[Dr. Reid, of the New York Hospital, has recently called public attention to the dangerous use of lead by confectioners, as a coloring matter for their candies, sugar plums, &c. as illustrated in an example detected by Dr. Colton, the House Physician. But the far greater probability that the sugar of which these articles are made, is itself poisonous, by reason of the lead often used in refining it, is worthy of chemical scrutiny. For if the lead exists in refined sugar here as in Europe, its extensive and universal use may render the inquiries into lead disease of still greater importance.

The following article may throw some light on this subject.]

The subjoined letter, recently addressed by Mr. Herapath, of Bristol, to the Editor of the *Times*, is well calculated to show the subtile nature of lead poison, even when it exists in the very insoluble form of carbonate of lead.

"Sir.—Your number of yesterday, in some remarks upon Dr. Scoffern's patent for purifying sugar by sulphurous acid, left the public in doubt as to what quantity of lead might be taken by human beings without injurious effects. Some time since, in the west of England, a river, the water of which had been used from time immemorial by the inhabitants of a village on its banks without injury, was found to affect their health; symptoms of indigestion abounded, with loss of flesh and appetite; and there were some few cases of colic; they believed that it arose from the use of the river water, as those who used water drawn from a spring at some distance were not so affected, I was requested to analyse the river water, and found in it one 500,000th part of carbonate of lead, which arose from a mine worked at the distance of three or four miles from the village, on the other side of a range of limestone hills.

"Your paragraph leaves it doubtful whether 1¼ grains of lead taken in a week would be injurious. In the case I relate there would be only one grain of lead in nine gallons of water ; and yet the health of the neighborhood was seriously affected. I am, Sir,

Yours respectfully, William Herapath.

"*Old Park, Bristol, Sept.* 12."

*** Allowing that the individuals poisoned by this water consumed as much as a gallon per day, or swallowed in some form or other the lead contained in that quantity of water, it follows that they would have taken no more than about *three-quarters of a grain* of lead per week! This infinite-issimel quantity, according to Mr. Herapath, was quite sufficient to endanger health. An observation of this kind clearly shows that we are not able to set a limit to the proportion of lead in an article of food consumed daily which will be inert or free from consequences injurious to health. If one *half-millionth part* of insoluble carbonate of lead may thus affect health, it is quite within the range of probability that this or a larger proportion of sulphite, or any other insoluble salt of lead, taken daily in sugar, treacle, or any article of food, may prove equally injurious. In reference to the sugar-refining question, Dr. Gregory asserts, that the highly *insoluble* carbonate of lead is *the truly poisonous* lead-compound; while he says it might be predicted of the sulphite of lead that it is as harmless as chalk, *because* of its excessive *insolubility*. This is an ingenious way of dealing with chemical doctrines. Either insolubility (in water) has nothing to do with the question of poisoning, or the carbonate of lead should be just as harmless as chalk,—an assumption which is contradicted by daily experience. It may be said that 500,000 parts of water will dissolve one part of carbonate of lead, this is highly probable; and, as insolubility is only a relative term, it is equally probable that this, or even a much smaller quantity of water, would dissolve one part of the sulphite of lead. The "excessively insoluble" sulphate of barytes is dissolved by 40,000 parts of water. It must be remembered, however, that the secretions of the stomach differ from water. They contain organic matters, acids, and salts, which act upon mineral substances in a way which chemical experiments on the solvent powers of *water* would fail to explain. Mr. Herapath has done good service by showing how minute a quantity of lead will effect human health; since facts of this kind will prevent a general trial of lead-refined sugar with the serious risk of endangering the health of the population.—*N. Y. Med. Gazette.*

